# Knowledge and uptake of occupational post-exposure prophylaxis amongst nurses caring for people living with HIV

**DOI:** 10.4102/curationis.v39i1.1593

**Published:** 2016-03-29

**Authors:** Lufuno Makhado, Mashudu Davhana-Maselesele

**Affiliations:** 1 Department of Nursing Sciences, North-West University, Mmabatho, South Africa

## Abstract

**Background:**

Nurses caring for people living with HIV (PLWH) are at higher risk of exposure to the human immunodeficiency virus (HIV) by needle sticks, cuts, getting body fluids in their eyes or mouth and skin when bruised or affected by dermatitis.

**Objectives:**

To determine knowledge, insight and uptake of occupational post-exposure prophylaxis (OPEP) amongst nurses caring for PLWH.

**Method:**

A cross-sectional descriptive design was used in this study. Stratified random sampling was used to sample 240 nurses. The study was conducted in a regional hospital in Limpopo province. Both parametric and non-parametric statistics were employed to analyse data.

**Results:**

A total of 233 nurses participated in the study. Sixty per cent (*n* = 138) of all nurses had a situation at work when they thought that they were infected by HIV and 100 (43%) nurses had experienced the situation once or more in the past 12 month. Approximately 40% did not know what PEP (post-exposure prophylaxis) is, and 22% did not know or were not sure if it was available in the hospital. Only few participants (*n* = 68, 29%) had sought PEP and most (*n* = 37, 54%) of them did not receive PEP when they needed it. There was a significant association between the knowledge and availability of PEP (*r* = 0.622).

**Conclusion:**

The study recommend an urgent need for policy makers in the health sector to put in place policies, guidelines and programmes that will rapidly scale up PEP services in health care settings, so that preventable occupationally acquired HIV infection can be minimised amongst nurses.

## Introduction

The rise of human immunodeficiency virus (HIV) infections nowadays had greatly exposed the health care settings to frequent occupational hazards that posed an amplified challenge to health care providers. Occupational exposure to HIV in either hospital or community health care settings presents a potential risk of infection (Hamlyn & Easterbrook 2007:329). Nurses, as the forefront health care providers, are perceived to be the most susceptible group with regard to occupational health hazards and work in fear of contagion with HIV.

The prescription of antiretroviral therapy (ART) as post-exposure prophylaxis (PEP) following exposure to HIV has now become routine and it is of paramount importance that individuals with potential risk of exposure are aware of the procedures to follow and where the first point of contact should be if an incident occurs (Hamlyn & Easterbrook 2007:329). PEP is short-term antiretroviral treatment to reduce the likelihood of HIV infection after occupational exposure.

### Problem statement

Although occupational exposure to HIV is preventable, it continues to impose risk on nurses and impact negatively on their health. Nurses caring for people living with HIV (PLWH) are at a greater risk of exposure to HIV by needle sticks or cuts, getting blood or other body fluids in their eyes or mouth and blood or other body fluids on their skin when it is chapped, scraped, or affected by dermatitis. It is of paramount importance that the awareness and knowledge about OPEP be sought amongst nurses caring for PLWH. Hence, this study sought to determine the knowledge and uptake of OPEP amongst nurses caring for PLWH.

#### Aim of the study

The purpose of this study was to determine knowledge and uptake of OPEP amongst nurses caring for PLWH.

#### Background

Globally, there are approximately 3 million HIV exposures amongst health care providers every year estimating to result in 200 to 5000 HIV infections (World Health Organisation [WHO] 2006). Occupational HIV exposure impacts heavily on the already burdened workforce with increased morbidity and mortality amongst nurses who provide care to PLWH.

The standard universal precaution guidelines (WHO 2006) had been in place for the last two decades, and stipulated the provision of adequate sharps containers, the training of workers at risk and prevention of transmission of blood-borne viruses, the use of gloves and eye wear, and safer devices such as needles that sheath or retract after use. These were said to have led to a significant reduction in needle prick, infection risks and other injuries (Beekmann & Henderson 2005). The Pruss-Ustun, Rapiti and Hutin’s Report (2003) stated that the global burden of disease resulting from sharps injuries reveals a number of factors which may contribute to their occurrence, including the use of unnecessary needles, the lack of availability of safer needle devices and sharps disposal containers, the lack of access to or failure to use sharps containers immediately after a procedure, and the continued recapping of needles after use.

Hamlyn and Easterbrook (2007:334) reported that regardless of occupational exposures being preventable, this continues to impose risk on nurses and impacts negatively on their health. Nurses caring for PLWH are at a greater risk of exposing themselves to HIV by the risky engagement they are faced with in the workplace, that is needle sticks or cuts, blood or other body fluids spills or splashes in their eyes, nostrils or mouth and exposed blood or other body fluids on their chapped or scraped skin, or affected by dermatitis.

Furthermore, it is of paramount importance that awareness and knowledge about OPEP be created amongst nurses caring for PLWH. This is based on limited information about the use of universal precautions by nurses in a resource-limited country such as South Africa. Universal precautions focus specifically on the prevention of exposure to blood and body fluids. The study sought to determine the awareness, knowledge and uptake of OPEP amongst nurses caring for PLWH in Vhembe district, Limpopo province, South Africa.

#### Research objectives

The objectives of the study were to:

- Determine the level of exposure to HIV amongst nurses caring for PLWH.
- Determine the awareness and knowledge about OPEP.
- Determine the uptake of PEP amongst nurses exposed.
- Establish the relationship between awareness and availability of OPEP in the unit.

#### Definition of concepts

**Occupational post-exposure prophylaxis**: The prophylaxis taken when a nurse working in a health care setting is potentially exposed to material infected with HIV.

**Occupational exposure**: Reasonably anticipated skin, eye, mucous membrane, or parenteral contact with blood or other potentially infectious materials, in this case HIV, that may result from a nurse’s duties.

#### Contribution to field

The findings of this study may help policy makers and programme planners in developing policies and programmes that will support awareness, knowledge and skills development regarding prevention and management of occupational exposure to HIV, as well as the uptake of OPEP, and promote a positive working environment.

### Literature review

Graziano (2010) defines occupational exposure as any contact with an infectious body fluid as a result of an injury with a needle or any other sharp instrument, or via mucous membranes or an existing cutaneous condition (wound, eczema, scratch, etc.). This applies to nurses caring for PLWH and patients with an unknown HIV status. Occupational exposure may place nurses at risk of HIV infection through injuries such as those involving a potentially contaminated needle or sharp instrument or chapped, abraded skin or contact with mucous membranes. A potentially infectious body fluid that comes from a person who carries an infection is termed infectious. Potentially infectious body fluids include blood, cerebrospinal fluid (CSF), synovial fluid, pleural fluid, pericardial fluid, amniotic fluid, semen, or vaginal secretions (Graziano 2010).

PEP comprises of administering a short course of ART to decrease the possibility of sero-conversion following events with high risk of exposure to HIV (National AIDS Control Organisation [NACO] 2009). The process that nurses have to follow after exposure involves first aid, counselling, risk assessment, relevant laboratory investigations with the consent of the exposed individual and source, followed by provision of a short course of ART for 28 days, and monitoring (Mathewos *et al*. 2013:508; NACO 2009).

PEP has been effective and able to prevent about 81% of seroconversion and is at present the only means of reducing the risk of HIV infection after exposure (Gupta *et al*. 2008:2). Unsafe practices such as the re-use of inadequately sterilised needles, careless handling of contaminated needles, and poor hazardous waste management, have the potential to increase the risk of acquiring blood-borne pathogens (Gupta *et al*. 2008:7; Sagoe-Moses *et al*. 2001:538). In 2005 about 3 million percutaneous occupational exposures to blood or other bodily fluids occurred in health care settings, the majority (90%) in developing countries (Gupta *et al*. 2008:2). The average risk of HIV transmission after a percutaneous exposure to HIV-infected blood has been estimated to be approximately 0.3% (Hoffmann, Bucholz & Schnitzler 2013). The risk after a mucous membrane exposure is approximately 0.09%. Although episodes of HIV transmission after non-intact skin exposure have been documented, the average risk for transmission by this route has not been precisely quantified but is estimated to be less than the risk for mucous membrane exposures (Kuhar *et al*. 2013:877). The risk for transmission after exposure to fluids or tissues other than HIV-infected blood also has not been quantified but is probably considerably lower than for blood exposures (Kuhar *et al*. 2013:877). This projects that the risk of acquiring blood-borne pathogens is high in Africa, especially in South Africa most probably reflecting the high prevalence of HIV.

There have been a number of studies conducted in the African continent regarding knowledge of PEP. Some studies have reported favourable knowledge of PEP amongst health care workers (Mathewos *et al*. 2013; Sarah *et al*. 2014), but several others have found rather important knowledge gaps on PEP amongst health care workers. In Nepal, only 6% of nurses in Chitwan Medical College Teaching Hospital had good knowledge on PEP, whilst in Zimbabwe 65% of health care workers and 83% in Ethiopia had poor knowledge on PEP (Jharna, Bijay & Kalpana 2012; Monera & Ncube 2012). Furthermore, amongst the exposed respondents, 81.6% did not use PEP, with 33.8% reporting lack of knowledge on the use of PEP (Tebeje & Hailu 2010). Similarly, inadequate knowledge on PEP has been reported amongst medical doctors in a tertiary hospital in Nigeria (Esin *et al*. 2011). Most studies suggest that nurses are at higher risk of occupational acquisition of HIV through needle stick injuries and contact with infected body fluids (Jharna *et al*. 2012; Owino; Srivanichakorn & Thepthien 2013). Therefore, it is of paramount important for nurses to have adequate knowledge on how they can protect themselves.

It was deemed of paramount importance to investigate the knowledge and uptake of PEP in a South African context given the heavy burden placed at nurses caring for PLWH given their increased health care access. This is carried out through HIV counselling and testing (HCT), prevention of mother to child transmission (PMTCT) and nurse initiated management of antiretroviral therapy (NIMART) to name a few, which are inclusive of procedures that expose nurses to infected fluids through needle pricks, fluid splash during obstetric care, and contact with an infected person’s skin when skin is cracked.

## Methods

### Design

A cross-sectional descriptive study was conducted in a regional hospital in Limpopo province.

### Context of the study

The study was conducted at one of the regional hospitals in Vhembe district, Limpopo province. Vhembe district is predominantly rural.

### Materials

The study focused on three nursing cadres (*N* = 233), namely enrolled nursing auxiliaries (ENA), enrolled nurses (EN) and professional nurses (PN) caring for PWLH. Purposive sampling was used in this study because of the nurses’ experience in caring for PLWH.

### Data collection methods

Data collection was attained using a self-administered questionnaire. The questionnaire comprised of two sections, i.e. demographics and a seven item questionnaire which incorporates measures for exposure and PEP uptake related questions. The questionnaire was prepared by selecting relevant studies carried out by Esin *et al*. (2011:465), Owolabi *et al*. (2011:180) and Agaba *et al*. (2012), which were modified according to the field experiences of the researchers. The questionnaire was then pre-tested amongst 13 nurses in a hospital and further modifications were incorporated with the aid of PEP experts; however, reliability analysis was not performed. Participants were recruited from the nurses’ respective wards at the hospital. An information sheet was provided to inform them about the purpose of the study after which the questionnaires were disseminated.

### Data analysis

Statistical package for social sciences (SPSS 21) was used to assemble and analyse the collected data. Frequencies and percentages were used to analyse the demographics as well as the different questions in the questionnaire and were tabulated. Linear correlation was carried out to establish possible relationship between availability of PEP and knowledge of PEP. The significance level was set at 0.01.

## Ethical considerations

Ethical clearance of the study was granted by the North-West University Ethics Committee, and permission to conduct the study by the Limpopo Department of Health. The study protected the rights and dignity of the participants, and they were informed that the research was of no harm to them. Voluntary participation was encouraged and participants were informed of their right to terminate their participation at any given point if they felt uncomfortable. Written consent of all participants was sought after detailed information about the research was given to participants. The researchers ensured that all collected data were stored in a locked place and electronic data were saved in a password-protected device to which only the researchers had access. No names of the participants and hospital were divulged.

## Results

Two hundred and thirty three (97%) of 240 questionnaires were returned completed and analysed. The study was inclusive of PNs (*n* = 109, 47%), ENs (*n* = 58, 25%), and ENAs (*n* = 66, 28%). Female nurses (*n* = 181, 78%) dominated the study (Table 1).

**TABLE 1:** Demographic characteristic.

Variable	PN (*n* = 109)	EN (*n* = 58)	ENA (*n* = 66)
**Age**		
21–30	40 (37%)†	0	22 (33%)
31–40	21 (19%)	27 (47%)†	19 (29%)
41–50	30 (28%)	21 (36%)	24 (36%)†
50 and above	18 (16%)	10 (17%)	1 (2%)
**Gender**		
Male	14 (13%)	11 (19%)	27 (41%)
Female	95 (87%)†	47 (81%)†	39 (59%)†
**Enrolled in any course presently**		
Not enrolled	99 (91%)†	32 (55%)†	64 (97%)†
Enrolled	10 (9%)	26 (45%)	2 (3%)
**Ward allocation**
OPD/Casualty	9 (8%)	6 (10%)	3 (4%)
Medical and palliative wards	41 (38%)†	22 (38%)†	23 (35%)
Surgical wards	20 (18%)	16 (28%)	25 (38%)†
ICU/OT/Private ward	13 (12%)	7 (12%)	14 (21%)
Maternity wards	26 (24%)	7 (12%)	1 (2%)

PN, professional nurses; EN, enrolled nurse; ENA, enrolled nursing auxiliaries.

† Denotes the highest %.

Sixty per cent of nurses (*n* = 136) experienced a situation at work where they were afraid they had been infected with HIV; this had happened to them once (*n* = 54, 23%), twice (*n* = 25, 11%) and more than five times (*n* = 21, 9%) in the past 12 months. It was revealed that 40% (*n* = 92) of nurses did not know what PEP is, and 22% (*n* = 51) did not know or were not sure if it was available in the hospital/unit they were working in. Only 68 (29%) nurses had sought PEP and the majority (*n* = 37, 54%) of them did not receive PEP when they needed it during their last experience.

The findings in Table 2 show that those who did not seek PEP or sought it but did not receive it, was because they did not need PEP (42%, *n* = 81), they did not have enough information about PEP (16%, *n* = 33), did not want to take an HIV test (2%, *n* = 3), PEP was not available (8%, *n* = 16), did not know where to go (12%, *n* = 25), and were afraid to go through the process (20%, *n* = 41).

**TABLE 2:** Responses to different questions in the questionnaire.

Variable	*n*	%
**Have you ever had a situation at work where you have been afraid you were infected with HIV? (*N* = 233)**		
Yes	136	60
No	92	40
**How many times has that happened in the last 12 months? (*N* = 233)**		
Never happened	133	57
Happened once	54	23
Happened twice	25	11
Happened five or more times	21	9
**Do you know what post-exposure prophylaxis (PEP) is? (*N* = 230)**		
Yes	138	60
No	92	40
**Is PEP available in the unit? (*N* = 233)**		
Yes	172	74
No	10	4
Do not know/not sure	51	22
**Have you ever sought PEP? (*N* = 233)**		
Yes, every time I needed it	36	15
Yes, some of the times I needed it	32	14
No, I have never sought it	165	71
**When u sought PEP last time, did you get it? (*N* = 68)**		
Yes	31	46
No	37	54
**If you have never sought PEP, did not seek it last time you needed it or sought it but somehow did not get it, what was the main reason for that? (*N* = 202)**		
Did not need it	84	42
Did not have enough information About PEP	33	16
Did not want to take the HIV test	3	2
PEP not available	16	8
Did not know where to go	25	12
Was afraid to go through the process	41	20

PEP, post-exposure prophylaxis.

Table 3 shows a significant association between the knowledge about PEP and availability of PEP (*r* = 0.622) in the units that nurses are working in, which suggests that the availability of PEP in the unit increases the knowledge about PEP.

**TABLE 3:** Correlation coefficients of knowledge about PEP and availability of PEP.

Knowledge about PEP		Correlation
Availability of PEP	Pearson correlation	0.622**
	Sig. (2-tailed)	0.000

PEP, post-exposure prophylaxis.

** Correlation is significant at the 0.01 level (2-tailed).

## Discussion

Nurses, as key players in the health care provision in South Africa, need education and awareness about PEP as an important factor for successful prevention of transmission of HIV within the workplace. Nurses showed to be highly exposed to HIV, and this increases their fear of contagion, as they are on a day to day basis faced with HIV patients or PLWH as well as patients with an unknown HIV status. This is in line with a Cameroonian study by Aminde *et al*. (2015) stating that a majority of the nurses (85%) considered themselves to be at risk of exposure to HIV at the workplace with about 68% admitting to have been exposed in the past. This indicates that nurses’ exposure is a serious problem and has an impact in the provision of care towards PLWH, as nurses continually care for PLWH in settings where universal precautions cannot be maintained because of a lack of resources.

The majority of nurses (60%) knew about PEP and this was in line with findings by Owolabi *et al*. (2011:181) revealing that a majority of the respondents amongst health care providers working at the University of Abuja Teaching hospital (UATH), Gwagwalada, were aware of PEP. However, the major concern was the fact that 40% did not know anything about PEP. Ignorance in this area of work can have a disastrous outcome on the health of nurses (Sabane, Dixit & Durge 2011:29). It is safe to assume that this will also undermine the confidence of the nurses to deal with patients confidently and effectively (Sabane *et al*. 2011:29). According to Ooi, Dayan and Yee (2004), low levels of awareness and knowledge of HIV and PEP may translate to missed opportunities for access to PEP, and potential HIV infections.

Not all nurses caring for PLWH were aware of the availability of PEP in the unit they were working which is in line with the findings in the study by Ooi *et al*. (2004) that about 68.5% of those surveyed were aware of the availability of HIV PEP for high risk occupational exposures. Awareness of availability of PEP may provide, and increase, confidence of nurses caring for PLWH and reduce fear of HIV contagion.

Regardless of the high number of exposure amongst nurses, it was evident that only a few sought PEP. Owolabi *et al*. (2011:181) reported high exposure rate amongst the study respondents with only about 6% seeking PEP which support these findings. Furthermore, this finding is supported by findings from a similar study conducted in Nigeria that revealed poor attitudes of health care providers toward PEP (Obi, Waboso & Ozumba 2005:372). The study revealed poor seeking of PEP which may have been the result of a lack of knowledge, as well as the different reasons that this study has revealed.

The following reasons were mentioned by nurses as barriers to seeking PEP: they did not want to test for HIV; PEP was not available in the unit/hospital; and nurses did not want to go through the process of PEP. Fear by some nurses of going through the process of PEP seemed to be a barrier to taking the prophylaxis, which may be the greatest factor of nurses’ morbidity and mortality as a result of HIV infection in a workplace. According to Abrahams and Jewkes (2010) the combination of feeling ill from the medication and the association of the drugs with AIDS was said to create immense distress and was a barrier to taking PEP. The physical effect of ARVs which includes side effects that are well known by these nurses from their own patients, increases their fear of going through the PEP process. The mentioned reasons are in line with Abrahams and Jewkes (2010), stating that physical and side effects of the drugs clearly exacerbated the fear of HIV infection and this should be taken as a potential barrier to PEP adherence.

Other nurses revealed not having enough information about PEP, not knowing where to go for PEP, as well as not needing it which may be because of the fact that the source had tested negative. These findings were supported by Owolabi *et al*. (2011:181) stating that the reason why many of them did not need PEP was that the patient was HIV negative.

These findings reveal the fact that not all nurses are aware or knowledgeable of the importance of PEP in their unit/hospital and the preventive necessity thereof. However, if all these nurses who sought PEP and not receive it seroconvert to HIV infection, it can have severe implications for the health care system. The health care system is prone to suffer from nursing workforce morbidity and mortality as a result of occupational acquired HIV infections. This will correspondingly have a negative impact in the South African nursing profession as this may induce fear of becoming a nurse. If nurses acquire HIV occupationally it will definitely discourage other health care providers caring for PLWH (Owolabi *et al*. 2011:182)

### Practical implications

The findings of this study are of great use to make recommendations to guideline and policy developers, who may utilise the information to develop policies, guidelines and programmes that will assist in strengthening and promoting universal precautions, and the uptake of OPEP.

### Limitations

The findings of the study cannot be generalised to other regional hospitals in the province as only one regional hospital was used.

## Recommendations

As a result of the research findings, the following recommendations are made.

### Practice

- Emphasis on universal precaution and infection control in hospitals needs an improved approach.
- It is, therefore, a necessity that nurses caring for PLWH should be informed about PEP guidelines and policies; this includes information about potential risk of exposure, importance of early reporting following exposure, strict avoidance of exposure and readily available first aid kits.
- There is a crucial necessity for policy makers in the health sector to set in place policies, guidelines and programmes that will quickly measure up/monitor and evaluate PEP services in health care settings, so that escapable occupationally attained HIV infection can be avoided amongst our nurses.
- Organisational provision of all protective equipment must be a priority.
- PEP induction training/and refresher courses are required as they form an integral part of the initial training of health PN on universal precaution measures.

### Future research

- A larger study over a diversity of nurses exploring their attitude, perceptions and experiences with regard to OPEP is required.
- Epidemiologic studies on the prevalence of PEP uptake in South Africa amongst exposed nurses are required.
- Evaluate OPEP programme.

## Conclusion

Not all nurses exposed to HIV are aware of PEP. It was also evident that not all exposed nurses sought PEP and those who sought PEP did not all receive it when needed. Furthermore, some nurses revealed that they never sought PEP because they did not want to go through its process, did not want to undergo the HIV test, did not know where to go for it, and did not have sufficient information on PEP. There was a significant association between availability and knowledge of PEP in the unit. Although HIV is regarded as a chronic disease, strict preventive measures are still a priority and these measures need to be taken seriously to promote and maintain a healthy nursing workforce in South Africa.
